# Release of c-FLIP brake selectively sensitizes human cancer cells to TLR3-mediated apoptosis

**DOI:** 10.1038/s41419-018-0850-0

**Published:** 2018-08-29

**Authors:** Lugain Alkurdi, François Virard, Béatrice Vanbervliet, Kathrin Weber, Florent Toscano, Marc Bonnin, Nolwenn Le Stang, Sylvie Lantuejoul, Olivier Micheau, Toufic Renno, Serge Lebecque, Yann Estornes

**Affiliations:** 1 Univ Lyon, Université Claude Bernard Lyon 1, INSERM 1052, CNRS 5286, Centre Léon Bérard, Centre de recherche en cancérologie de Lyon, F-69373 Lyon, France; 2 Univ Lyon, Université Claude Bernard Lyon 1, Faculté d’Odontologie, Hospices Civils de Lyon, INSERM 1052, CNRS 5286, Centre Léon Bérard, Centre de recherche en cancérologie de Lyon, F-69373 Lyon, France; 3 Département de Biopathologie – Registre MESONAT, Centre Léon Bérard, 69008 Lyon, U1086 INSERM-UCBN « Cancer & Prévention », Caen, France; 4 Département de Biopathologie, Centre Léon Bérard, 69008 Lyon, INSERM U823, Institut A. Bonniot, 38700 La Tronche, France; 5 Univ. Bourgogne Franche-Comté, INSERM, LNC UMR866, F-21000 Dijon, France; 6 Hospices Civils de Lyon, Centre Hospitalier Lyon-Sud, Service d’Anatomie Pathologique, 69495 Pierre Bénite Cedex, France

## Abstract

Toll-like receptor 3 (TLR3) mediates innate immune responses by sensing viral dsRNA, but also induces apoptosis selectively in cancer cells. Our analysis by immunohistochemistry revealed that TLR3 is frequently overexpressed in 130 non-small cell lung cancer (NSCLC) patients’ samples compared with normal bronchial epithelium (*P* < 0.0001, Mann–Whitney test), supporting the therapeutic potential of TLR3 ligand for this type of cancer. However, a proportion of TLR3-expressing cancer cell lines, including NSCLC, remain resistant to TLR3-mediated apoptosis, and the underlying mechanism of resistance remains unclear. We here investigated the molecular basis conferring resistance to non-transformed vs. transformed cells against TLR3-mediated cell death. In non-transformed epithelial cells cellular FLICE-like inhibitory protein (c-FLIP) and cellular Inhibitor of APoptosis (cIAPs) ubiquitin ligases exerted an efficient double brake on apoptosis signaling. In contrast, releasing only one of these two brakes was sufficient to overcome the resistance of 8/8 cancer cell lines tested. Remarkably, the release of the c-FLIP, but not cIAPs, brake only results in the sensitization of all human cancer cells to TLR3-mediated apoptosis. Taking advantage of the difference between transformed and non-transformed cells, we developed a rational strategy by combining the chemotherapeutic agent paclitaxel, which decreases c-FLIP expression, with TLR3 ligand. This combination was highly synergistic for triggering apoptosis in cancer cells but not in non-transformed cells. In vivo, the combination of paclitaxel with dsRNA delayed tumor growth and prolonged survival in a mouse xenograft lung tumor model. In conclusion, combining the release of the c-FLIP brake with TLR3 ligand synergizes to selectively kill cancer cells, and could represent an efficient and safe therapy against TLR3-expressing cancers such as NSCLC.

## Introduction

Toll-like receptor 3 (TLR3) is an endosomal pattern-recognition receptor that detects viral dsRNA, but also mRNA and small nuclear RNA released by damaged tissues. TLR3 mediates an innate immune response characterized by the production of type I IFNs and pro-inflammatory cytokines^1^. TLR3 signals through TIR domain-containing Adapter Molecule 1 (TICAM 1 also called TRIF) which allows the recruitment of TNF Receptor-Associated Factor (TRAF)-6, Receptor Interacting Protein kinase (RIPK)-1, and TRAF3 for the activation of NF-κB, MAPK, and IRF3 inflammatory signaling pathways^1^.

Besides the inflammatory response, we and others have reported that TLR3 ligands can induce apoptosis in various human tumor cells such as breast adenocarcinoma (ADC)^2^, clear renal carcinoma^3^, oral carcinoma^4^, head and neck cancer^5,6^, nasopharyngeal carcinoma^7,8^, melanoma^9,10^, prostate carcinoma^11^, multiple myeloma^12^, or non-small cell lung cancer (NSCLC)^13^. TLR3-mediated apoptosis in human cancer cells involves a signalosome called ripoptosome^13,14^. This death-signaling platform contains RIPK1/FADD/caspase-8/cIAPs/c-FLIP wherein RIPK1 plays a key scaffold function linking TLR3/TRIF to the caspase-mediated apoptotic machinery^13,14^. Hence, TLR3 activation engages the caspase-8-dependent “extrinsic” pathway of apoptosis, which is typically triggered by activation of the death receptors of the Tumor Necrosis Factor Receptor (TNFR) family^15^. In the condition of caspase-8 inhibition, death receptors as well as TLR3 can induce another form of regulated cell death, called necroptosis, with features of necrosis^16^. Necroptosis relies on the key components RIPK1, RIPK3, and mixed lineage kinase domain-like (MLKL) for the formation of a cytosolic death signaling platform called necrosome^17–20^. TLR3-mediated necroptosis has been mainly reported in transformed and non-transformed murine cells upon exposure to Poly(I:C) in the condition of caspases inhibition by Z-VAD compound^21–26^. The role of RIPK3 and MLKL has been well demonstrated for TLR3-mediated necrosis, but the requirement of RIPK1 remains controversial^22,23,25,26^.

Considering that induction of inflammatory pathways is a general outcome of TLR3 activation, the fact that TLR3-mediated apoptosis occurs in human tumor cell lines but not in their normal counterparts indicates that sensitivity is acquired during cell transformation. However, the molecular determinants of the differential sensitivity of transformed vs. non-transformed cells remain to be clarified. Cellular Inhibitor of APoptosis (cIAPs) ubiquitin ligases are negative regulators of TLR3 apoptotic signaling^7,8,10,13,14,27^. Consequently, combination of smac mimetics that trigger the proteasomal degradation of cIAPs with TLR3 ligands has been proposed as a treatment for melanoma and nasopharyngeal carcinoma^7,8,10^. cIAPs probably act by mediating RIPK1 poly-ubiquitylation, hence limiting the formation or stabilization of the ripoptosome^7,8,10,13,14,27^, as reported downstream of TNFR1^28,29^. Another well-known negative regulator of caspase-8-mediated apoptosis downstream of the death receptors is the anti-apoptotic protein cellular FLICE-like inhibitory protein (c-FLIP). Indeed, zymogen monomeric caspase-8 possesses a stronger affinity for c-FLIP long isoform (c-FLIP_L_) than for itself, therefore forming preferentially c-FLIP_L_/caspase-8 heterodimers^30^. These heterodimers retain a catalytic activity limited to proximal substrates, including RIPK1^31,32^, but do not mediate apoptosis. Consequently, recruitment of c-FLIP_L_ not only prevents the full pro-apoptotic activation of caspase-8 downstream TLR3 but would also destabilize the ripoptosome^14^.

Here we demonstrate the differential dependency on cIAPs and c-FLIP for the resistance of non-transformed vs. transformed cells to TLR3-mediated apoptosis. We found that the resistance of non-transformed cells is controlled by a double brake imposed by cIAPs and c-FLIP whereas the release of only one of these two brakes was sufficient to overcome the resistance of all cancer cell lines tested. Remarkably, the release of the c-FLIP brake only results in the sensitization of 8/8 human cancer cell lines but not normal cells to TLR3-mediated apoptosis. We took advantage of this difference to design a rational combination of TLR3 ligand with paclitaxel chemotherapy that suppresses the expression of c-FLIP in cancer cells. Paclitaxel synergized both in vitro and in vivo with TLR3 ligand to selectively kill tumor cells while sparing non-transformed cells.

## Results

### Human lung cancers frequently overexpress TLR3

**Fig. 1:**
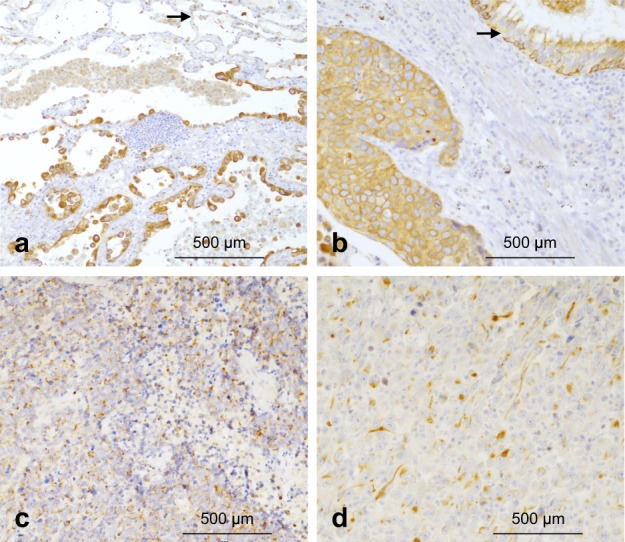
TLR3 is frequently overexpressed in human lung adenocarcinoma and squamous cell carcinoma. **a** Lepidic adenocarcinoma (ADC) presenting a strong cytoplasmic TLR3 staining, whereas adjacent normal alveolar cells (arrow) are not stained (with the exception of non-specific cytoplasmic staining of the macrophages). **b** Squamous cell carcinoma (SCC) with a diffuse and strong cytoplasmic TLR3 staining with membrane reinforcement, while TLR3 staining in adjacent normal bronchiolar epithelium (arrow) is less intense. **c** Small cell lung carcinoma (SCLC) with a weak granular cytoplasmic TLR3 staining. **d** Spindle cell sarcomatoid carcinoma (SC) with a weak TLR3 cytoplasmic staining. Original magnification × 200 (internal scales). Scale bars: 500 μm

Expression of TLR3 by tumor cells was reported to be a good biomarker for clinical response of breast cancer patients to TLR3 ligand Poly(A:U)^33^. We have now developed an automated immunostaining protocol and evaluated TLR3 expression in a cohort of human lung cancers. One hundred and thirty-nine lung cancer specimens, including 53 ADC, 47 squamous cell carcinoma (SCC), 30 sarcomatoïd carcinoma (SC), and nine small cell lung carcinoma (SCLC), were analyzed for TLR3 expression by immunohistochemistry (Table 1). Normal bronchial epithelium was weakly stained (median score of 50) with a discrete apical granular staining while alveolar cells remained negative (Fig. 1a, b). Remarkably, 52 out of 53 ADC (98%) and 43 out of 47 SCC (91%) were TLR3 positive and reached a median score of 100 and 90 that were both significantly higher than for normal bronchial epithelium (*P* < 0.0001) (Table 1, and Fig. 1a, b). Conversely, 5 out of 9 (60%) SCLC and 18 out of 30 (60%) SC expressed TLR3 but at a much lower level (median scores of 10 for both, *P* < 0.0001), indicating that either TLR3 is lost in these types of cancers or that they arise from a different cell of origin (Table 1, and Fig. 1c, d). Hence, as reported for other types of cancers (clear renal cell carcinoma^3^, oral squamous carcinoma (OSC)^34^, breast cancers, and melanoma^33^), NSCLC frequently overexpresses TLR3 compared to the normal bronchial epithelium, which warrants further investigation on targeting TLR3 in these cancers.

**Table 1:** TLR3 expression in human lung cancers

No. of cases studied	ADC *n* = 53	SCLC *n* = 9	SC *n* = 30	SCC *n* = 47	Comparison test
No. of pos cases (%)	52/53 98%	5/9 60%	18/30 60%	43/47 92%	*P* < 0.0001
Median scores	100	10	10	90	*P* < 0.0001
[Ranges]	[0–240]	[0–240]	[0–180]	[0–270]	

One hundred and thirty-nine lung cancers, including 53 adenocarcinoma (ADC) classified according to the new 2015 WHO classification of lung tumors in: 17 papillary, 16 acinar, 15 solid, two lepidic, one mucinous invasive and one micropapillary, 47 squamous cell carcinoma (SCC), 30 sarcomatoid carcinoma (SC), and nine small cell lung carcinoma (SCLC) were all analyzed by immunohistochemistry. Median score was calculated as described in the “Material and Methods”

### Release of cIAPs or c-FLIP brake promotes sensitivity of human cancer cells to TLR3-mediated apoptosis

**Fig. 2:**
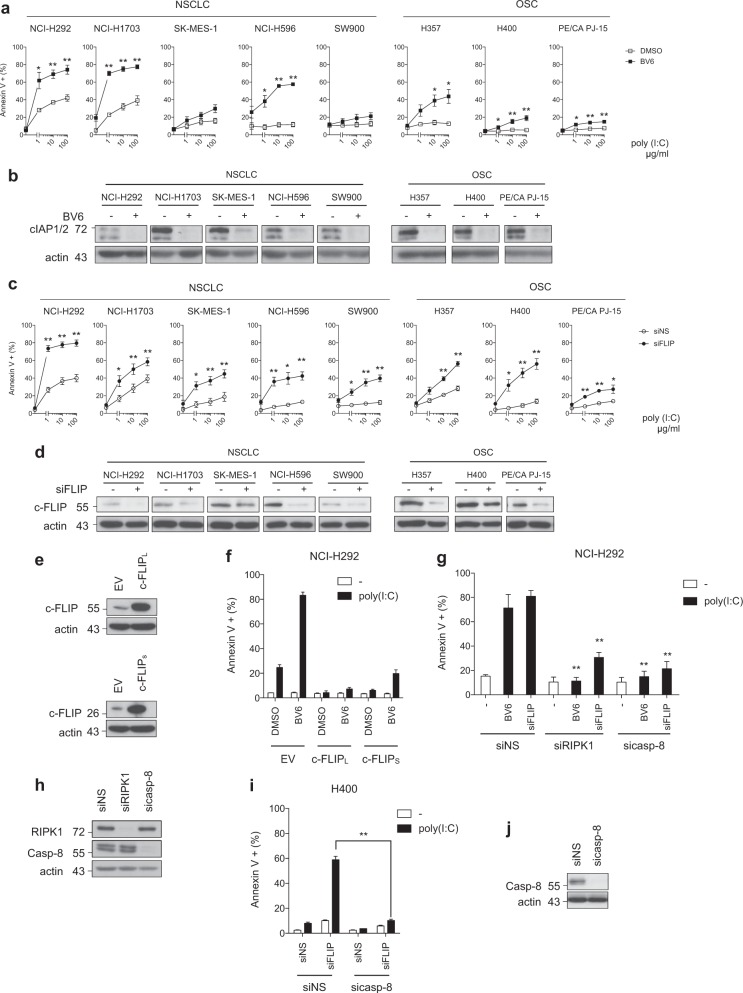
Releasing the cIAPs or c-FLIP brake overcomes the resistance of cancer cells to TLR3-mediated apoptosis. **a** Cancer cells were treated with smac mimetic BV6 (5 μM) for 1 h and then exposed to increasing doses of Poly(I:C) (1, 10, and 100 μg/ml) for 6 h. The percentage of Annexin V+ cells was determined by flow cytometry. Error bars represent S.E.M. of at least three independent experiments. *P < 0.05 and **P < 0.01. **b** Cancer cells treated for 1 h with BV6 were lysed and immunoblotted as indicated. **c** Cancer cells were transfected with a control non-silencing siRNA (siNS) or targeting c-FLIP (siFLIP), and then exposed to increasing doses of Poly(I:C) for 6 h. The percentage of Annexin V+ cells was determined by flow cytometry. Error bars represent S.E.M. of at least three independent experiments. *P < 0.05 and **P < 0.01. **d** Cancer cells transfected as in **c** were lysed and immunoblotted as indicated. **e** Analysis by western blot of c-FLIP_L_ and c-FLIP_S_ ectopic expression in NCI-H292 cells. **f** NCI-H292 cells stably overexpressing c-FLIP_L_ or c-FLIP_S_ isoform were treated with BV6 for 1 h and then exposed to Poly(I:C) (100 μg/ml) for 6 h. The percentage of Annexin V+ cells was determined by flow cytometry. Error bars represent S.E.M. of two independent experiments. **g** NCI-H292 cells were transfected with control siRNA (siNS) or targeting RIPK1 (siRIPK1) or caspase-8 (sicasp-8), and then further transfected with siFLIP or treated with BV6 for 1 h. The cells were exposed to Poly(I:C) (100 μg/ml) for 6 h, and the percentage of Annexin V+ cells was determined by flow cytometry. Error bars represent S.E.M. of three independent experiments. **P < 0.01 vs. corresponding conditions in NS siRNA treated cells. **h** NCI-H292 cells transfected as in **g** were lysed and immunoblotted as indicated. **i** H400 cells were transfected with siNS or sicasp-8, and then further transfected with siFLIP. The cells were then treated with Poly(I:C) (100 μg/ml) for 6 h, and the percentage of Annexin V+ cells was determined by flow cytometry. Error bars represent S.E.M. of three independent experiments. **P < 0.01. **j** H400 cells transfected as in **i** were lysed and immunoblotted as indicated

We have previously reported that cIAPs can inhibit the induction of apoptosis by TLR3 synthetic ligand Poly(I:C) in two NSCLC cell lines, NCI-H292 and NCI-H1703 cells^13^. To extend our observations we analyzed three additional NSCLC and three OSC cell lines that all produce cytokines/chemokines in response to Poly(I:C) (Supplemental Fig. 1), suggesting the presence of a functional TLR3 signaling pathway. However, none of these six cell lines showed significant sensitivity to Poly(I:C) alone (Fig. 2a). Pretreatment with the smac mimetic BV6 that triggers the proteasomal degradation of cIAPs^35^ greatly sensitized two cell lines (NCI-H596 and H357), and increased the percentage of NCI-H292 and NCI-H1703 cell death (Fig. 2a, b). Therefore, smac mimetic/Poly(I:C) combination can enhance the death of cancer cells but the effects remain limited to a proportion of cell lines.

c-FLIP is a classical negative regulator of caspase-8, which has also been previously reported to inhibit TLR3-mediated death^14^. In contrast to smac mimetic treatment, c-FLIP knockdown by siRNA transfection not only increased the level of Poly(I:C) cytotoxicity in the sensitive cell lines but also rendered all resistant cancer cell lines susceptible to TLR3-mediated death (Fig. 2c, d). To confirm that c-FLIP confers resistance to TLR3-mediated death, we overexpressed c-FLIP in NCI-H292 cells (Fig. 2e). Overexpression of c-FLIP_L_ or c-FLIP_S_ not only blocked Poly(I:C) cytotoxicity but also reverted the sensitization mediated by BV6 (Fig. 2f). This result confirms the crucial role of c-FLIP in the resistance against TLR3-mediated death, and suggests and cIAPs and c-FLIP molecular brakes exert their function along the same signaling pathway. Accordingly, the sensitization of NCI-H292 cells to Poly(I:C) cytotoxicity by releasing either the cIAPs or the c-FLIP brake was greatly decreased by RIPK1 knockdown, and prevented by caspase-8 knockdown (Fig. 2g, h), indicative of apoptosis. The requirement of caspase-8 for Poly(I:C)-induced apoptosis was confirmed in H400 cells sensitized to death by cFLIP siRNA (Fig. 2i, j). Taken together, these results indicate that single inhibition of either cIAPs or c-FLIP can sensitize cancer cells to TLR3-mediated, RIPK1/caspase-8-dependent apoptosis.

### cIAPs and c-FLIP exert an efficient double brake against TLR3-mediated apoptosis in normal bronchial epithelial cells

**Fig. 3:**
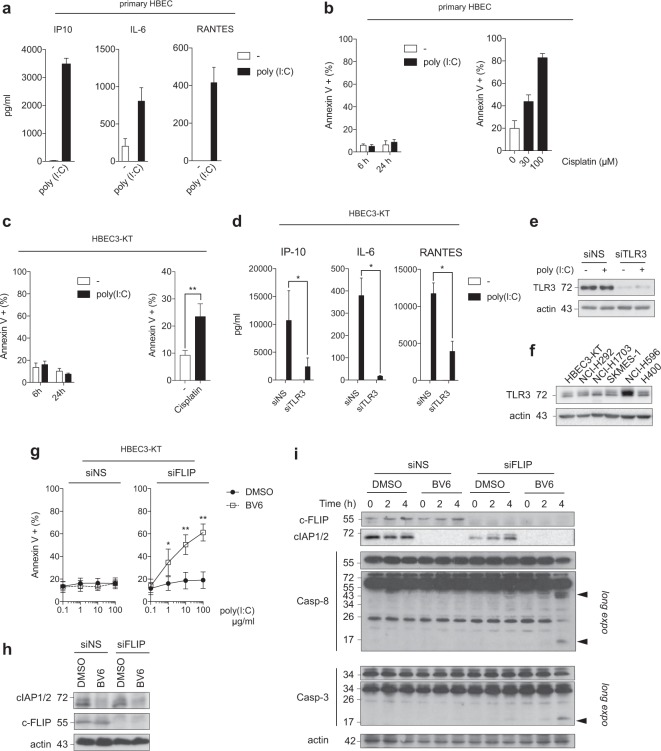
cIAPs and c-FLIP exert an efficient double brake against TLR3-mediated apoptosis in normal bronchial epithelial cells. **a** Primary human bronchial epithelial cells (HBEC) were exposed to Poly(I:C) (100 μg/ml) for 24 h, and the secretion of chemokines/cytokines were determined. Error bars represent S.E.M. of two independent experiments. **b** Primary HBEC were exposed to Poly(I:C) (100 μg/ml) for 6 and 24 h (left panel), or to cisplatin for 24 h (right panel), and the percentage of Annexin V+ cells was determined by flow cytometry. Error bars represent S.E.M. of three (left panel) and two (right panel) independent experiments. **c** Human immortalized bronchial epithelial HBEC3-KT cells were treated with Poly(I:C) (100 μg/ml) for 6 h and 24 h (left panel), or to cisplatin (100 μM) for 24 h (right panel), and the percentage of Annexin V+ cells was determined. Error bars represent S.E.M. of four (left panel) and three (right panel) independent experiments. **P < 0.01. **d** HBEC3-KT cells were transfected with a control non-silencing siRNA (siNS) or targeting TLR3 (siTLR3), exposed to Poly(I:C) (100 μg/ml) for 24 h, and the secretion of chemokines/cytokines was determined by ELISA. Error bars represent S.E.M. of three independent experiments. *P < 0.05. **e** HBEC3-KT cells were transfected as in **d**, lysed, and immunoblotted as indicated. **f** The indicated cells were lysed and immunoblotted for TLR3 and actin. Representative images of three independent experiments. **g** HBEC3-KT cells transfected with a control siRNA (siNS) or targeting c-FLIP (siFLIP), and were treated for 1 h with BV6 (5 μM). The cells were then exposed for 6 h to increasing doses of Poly(I:C), and the percentage of Annexin V+ cells was determined. Error bars represent S.E.M. of four independent experiments. *P < 0.05 and **P < 0.01. **h** HBEC3-KT cells were treated as in **f**, lysed, and immunoblotted as indicated. **i** HBEC3-KT transfected with siNS or siFLIP were then exposed to Poly(I:C) (100 μg/ml) for the indicated times in presence or absence of BV6 (5 μM). The cells were then lysed and immunoblotted as indicated. Caspase cleavage products are indicated by arrowheads. Representative images of two independent experiments

In contrast to cancer cells, non-transformed airway epithelial cells appear to be resistant to TLR3-induced apoptosis in vitro^5,7^. Accordingly, primary human bronchial epithelial cells (HBECs) produce IP10, IL-6, and RANTES in response to Poly(I:C) but remain completely resistant to apoptosis (Fig. 3a, b). Moreover, the non-transformed HBEC3-KT cells, which are immortalized by ectopic expression of CDK4 and hTERT^36^, were also resistant to Poly(I:C)-induced apoptosis (Fig. 3c). Primary HBEC and HBEC3-KT cells were, however, sensitive to cisplatin chemotherapy cytotoxicity, indicating that non-transformed cells were not resistant to all kind of death stimuli (Fig. 3b, c). Poly(I:C) triggered the secretion of IP10, IL-6, and RANTES chemokines/cytokines, which was inhibited by TLR3 knockdown in HBEC3-KT cells (Fig. 3d, e). HBEC3-KT cells expressed similar level of the 72 kDa cleaved form of TLR3^37,38^ than NCI-H292, NCI-H1703, SK-MES-1, or H400 cancer cells, while NCI-H596 cells expressed higher level (Fig. 3f). This result indicates that the resistance to Poly(I:C) cytotoxicity of non-transformed HBEC-3KT, but also of cancer cells, cannot be explained by the level of TLR3 expression, and that TLR3 signaling toward apoptosis in non-transformed cells is blocked at a certain stage. Therefore, we then addressed the role of cIAPs and c-FLIP in the resistance of the non-transformed HBEC3-KT cells to TLR3-mediated apoptosis. In contrast to cancer cells, neither BV6 nor c-FLIP siRNA alone sensitized HBEC3-KT cells to Poly(I:C)-induced apoptosis (Fig. 3g, h). However, the resistance of HBEC3-KT cells to Poly(I:C) was abolished when BV6 was combined with c-FLIP siRNA (Fig. 3g, h). Accordingly, caspase-8 and caspase-3 cleavages in response to Poly(I:C) treatment were detected only when both cIAPs and c-FLIP brakes were released (Fig. 3i). These results indicate that TLR3 apoptotic signaling in HBEC3-KT cells is efficiently blocked by the cIAPs and the c-FLIP brakes. Sensitization of HBEC-3KT cells to Poly(I:C)-triggered death by BV6/c-FLIP siRNA combination was prevented by caspase-8 (Supplemental Fig. 2a, b) or RIPK1 knockdown (Supplemental Fig. 2c, d), and by Z-VAD pretreatment (Supplemental Fig. 2e), indicative of pure apoptosis. Accordingly, necrostatin-1 (nec-1, an inhibitor of the kinase activity of RIPK1 that blocks necroptotic cell death^39^) had no effect (Supplemental Fig. 2e). Altogether, these results suggest that TLR3-mediated, RIPK1/caspase-8-dependent apoptosis signaling in non-transformed HBECs is prevented by an efficient cIAPs/c-FLIP double brake.

### Paclitaxel induces c-FLIP downregulation, and sensitizes cancer cells to TLR3-mediated apoptosis

**Fig. 4:**
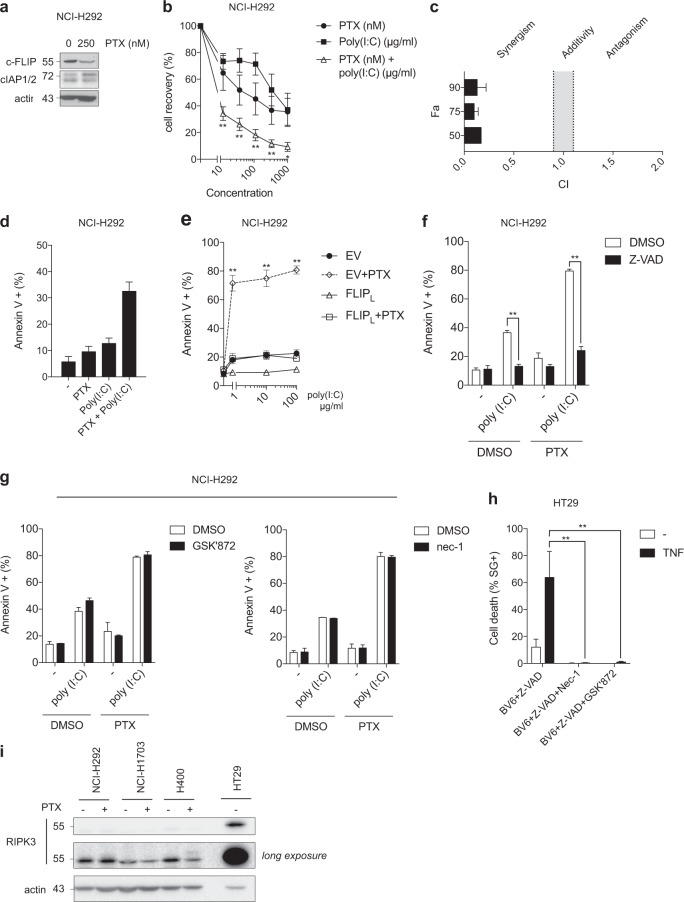
Paclitaxel induces c-FLIP downregulation, and sensitizes cancer cells to TLR3-mediated apoptosis. **a** Analysis by western blot of c-FLIP and cIAP1/2 levels in NCI-H292 cells treated with 250 nM paclitaxel (PTX) for 2 h, washed, and then incubated with medium for 24 h. **b** Viability curves of NCI-H292 cells treated or not with increasing doses (nM) of PTX for 2 h, washed, and 24 h later exposed or not to increasing doses of Poly(I:C) (μg/ml) for 2 h and then washed; 48 h later, cell survival was measured with MTS assay. Error bars represent S.E.M. of three independent experiments. *P < 0.05 and **P < 0.01 vs. Poly(I:C)-treated cells. **c** Combination index (CI) for a fraction affected (Fa) of 50, 75, or 90% of the drug association between PTX and Poly(I:C) for NCI-H292 cells and calculated using the method of Chou and Talalay^43^. Synergy is characterized by a CI < 0.9, additivity by a CI = 1 ± 0.1, and antagonism by a CI > 1.1. Error bars represent S.E.M. of three independent experiments. **d** Percentage of Annexin V+ NCI-H292 cells treated with 250 nM PTX as in **a**, and then exposed or not 24 h later to sub-optimal concentration of Poly(I:C) (0.08 µg/ml) for 6 h. Error bars represent S.E.M. of two independent experiments. **e** Percentage of Annexin V+ NCI-H292 cells overexpressing c-FLIP_L_ treated with 250 nM PTX as in **a**, and then exposed 24 h later with increasing doses of Poly(I:C) for 6 h. Error bars represent S.E.M. of three independent experiments. **P < 0.01 vs. PTX-treated EV cells. **f** and **g** NCI-H292 cells were treated with 250 nM paclitaxel as in **a**, and then exposed 24 h later with Poly(I:C) (10 µg/ml) for 6 h in absence (DMSO) or presence of Z-VAD (20 µM) (**f**), RIPK3 kinase inhibitor GSK'872 (2.5 µM) (**g**, left panel), or nec-1 (10 µM) (**g**, right panel). Error bars represent S.E.M. of three (**f**) and two (**g**) independent experiments. **P < 0.01. **h** HT29 cells were treated with BV6 (2.5 µM) + Z-VAD (20 µM) for 1 h, and then exposed or not to TNF (20 ng/ml) for 8 h. Cell death was measured using an Incucyte ZOOM® system analyzing the Sytox green positive (SG+) cells. **P < 0.01

We then reasoned that combining a TLR3 ligand with clinical compounds that downregulate c-FLIP expression could be a valuable strategy to selectively kill cancer cells while sparing non-transformed cells. Paclitaxel (Taxol) is a microtubule-stabilizing drug used for the treatment of various types of cancers, and has been previously reported to inhibit the expression of c-FLIP^40–42^. Accordingly, we found that c-FLIP was downregulated in both NCI-H292 and NCI-H1703 NSCLC cell lines treated with paclitaxel (Fig. 4a and Supplemental Fig. 3a). Of note, cIAPs level was not affected by paclitaxel treatment in NCI-H292 cells (Fig. 4a). Pretreatment with paclitaxel followed 24 h later by Poly(I:C) exposition for 48 h strongly decreased the long-term viability of NCI-H292 and NCI-H1703 cells compared to either drug used alone (Fig. 4b and Supplemental Fig. 3b). Based on these data, we calculated the combination index (CI) of the paclitaxel and Poly(I:C) association for a fraction affected of 50, 75, or 90% (using the method previously described by Chou and Talalay^43^), and revealed a strong synergy (CI < 0.5) for both cell lines (Fig. 4c and Supplemental Fig. 3c). In agreement, sub-optimal concentrations of paclitaxel (250 nM) and Poly(I:C) (0.08 µg/ml) induced robust cell death when both compounds were combined in NCI-H292 and NCI-H1703 cell lines (Fig. 4d and Supplemental Fig. 3d). Hence, paclitaxel mimics the effect observed when using c-FLIP siRNA (Fig. 2). Remarkably, paclitaxel-mediated sensitization to Poly(I:C) cytotoxicity was completely reverted in NCI-H292 cells overexpressing c-FLIP (Fig. 4e), suggesting that the effect of paclitaxel was indeed mediated through the decrease of c-FLIP. In agreement, in H400 cancer cells that can be sensitized to Poly(I:C)-triggered apoptosis when the c-FLIP, but not cIAPs, brake only is released (Fig. 2), paclitaxel also reduced the expression of c-FLIP (Supplemental Fig. 3e), and sensitized these cells to TLR3-mediated death (Supplemental Fig. 3f). However, we found that paclitaxel did not sensitized NCI-H596 and SKMES-1 cancer cells to Poly(I:C) cytotoxicity, but also did not downregulate c-FLIP in these cells (Supplemental Figs. 3h–3k). Together, these results indicate that paclitaxel can release the c-FLIP brake in cancer cells, which correlates with the sensitization to Poly(I:C) cytotoxicity.

We then investigated the relative contribution of apoptosis vs. necroptosis in the cell death induced by the paclitaxel/Poly(I:C) combination. First, we found that caspases inhibition by Z-VAD pre-treatment blocked the cytotoxicity of the paclitaxel/Poly(I:C) combination in both NCI-H292 (Fig. 4f) and H400 cells (Supplemental Fig. 3g). Then, inhibition of the kinase activity of RIPK1 or RIPK3 by nec-1 and GSK’872, respectively, did not affect the death induced by Poly(I:C) alone or by the paclitaxel/Poly(I:C) combination in NCI-H292 cells (Fig. 4g). In contrast, both nec-1 and GSK'872 prevented the death of HT29 human colon cancer cells treated by the TNF/BV6/Z-VAD necroptotic cocktail^44^ (Fig. 4h). RIPK3 expression is repressed in several types of cancers and cancer cell lines, which results in the inhibition of necroptosis^45,46^. Interestingly, we found that the protein level of RIPK3 in NCI-H292, NCI-1703, and H400, was extremely low compared to HT29 cells, and paclitaxel treatment did not modulate RIPK3 expression (Fig. 4i). Together, these results demonstrate that the paclitaxel/Poly(I:C) combination triggers apoptosis, but not necroptosis, in our cancer cellular models.

### Paclitaxel does not induce c-FLIP downregulation, and does not sensitize non-transformed cells to TLR3-mediated apoptosis

**Fig. 5:**
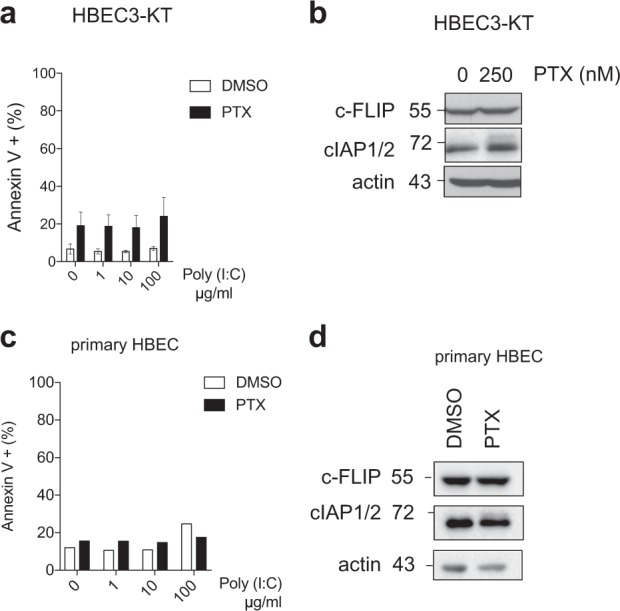
Paclitaxel does not induce c-FLIP downregulation, and does not sensitize non-transformed cells to TLR3-mediated apoptosis. **a** HBEC3-KT cells were treated with 250 nM paclitaxel (PTX) for 2 h, washed, and incubated with medium for 24 h. The cells were then exposed to increasing doses of Poly(I:C) for 6 h. Error bars represent S.E.M. of three independent experiments. **b** Analysis by western blot of c-FLIP and cIAP1/2 levels in HEBC3-KT cells treated with 250 nM PTX as in **a**. **c** Primary HBEC cells were treated with 250 nM PTX as in **a**, and then exposed 24 h later with increasing doses of Poly(I:C) for 6 h. One representative experiment out of two independent experiments is shown. **i** Analysis by western blot of c-FLIP and cIAP1/2 levels in primary HBEC cells treated with 250 nM PTX as in **a**

We then addressed the sensitivity to the paclitaxel/poly(I:C) combination of the non-transformed HBECT-3KT cells which are, in contrast to cancer cells, efficiently protected against TLR3-mediated apoptosis via a cIAPs/c-FLIP double brake. Remarkably, HBEC3-KT cells remained fully resistant to Poly(I:C) after pre-treatment with a dose of paclitaxel (250 nM) that substantially sensitize cancer cells (Fig. 5a). Unexpectedly, however, no detectable decrease of c-FLIP expression was observed in HBEC3-KT cells after treatment with 250 nM paclitaxel although it resulted in 20% cell death (Fig. 5a, b). The level of cIAPs was also not altered by paclitaxel treatment (Fig. 5b). Similar results were obtained with primary HBEC cells for which paclitaxel treatment did not sensitize to Poly(I:C)-induced death, and did not result in c-FLIP or cIAPs downregulation (Fig. 5c, d). Taken together, our data indicate that paclitaxel does not affect c-FLIP level in non-transformed cells, and does not sensitize them to TLR3-mediated apoptosis.

### Paclitaxel/Poly(I:C) combination therapy inhibits the growth of human NSCLC xenograft in mice

**Fig. 6:**
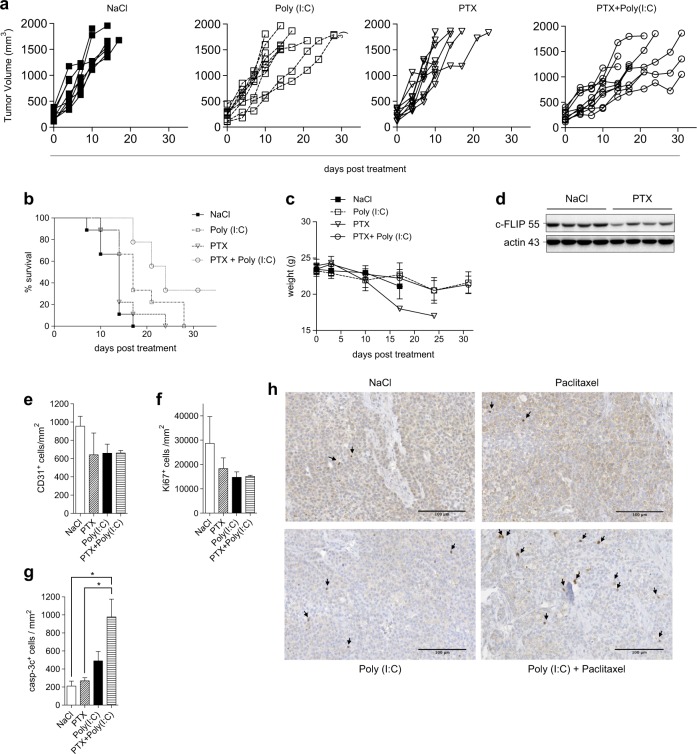
Combination of paclitaxel with Poly(I:C) triggers apoptosis in human NSCLC xenografts in mice and reduces the tumor growth. 1×10^6^ NCI-H292 cells were injected s.c. in nude mice. Once the tumors became detectable (day 0), mice were injected i.p. once a week wth NaCl, Poly(I:C) (0.1 mg/kg), paclitaxel (PTX) (10 mg/kg), or paclitaxel followed 24 h later by Poly(I:C) (nine mice/group). **a** Curves of the tumors growth monitored every 3 days. Data are representative of two independent experiments. **b** Kaplan–Meier survival curves. Mice were sacrificed when the tumors reached a volume >1800 mm^3^. Data are representative of two independent experiments. (Poly(I:C) vs. Poly(I:C) + PTX: *P* < 0.05; PTX vs. Poly(I:C) + PTX: *P* < 0.01). **c** Curves of the body weight of the mice. The measurements were started on the first day of treatment. At days 17 and 24, only one mouse was alive in the PTX group. Data are representative of two independent experiments. **d** Analysis by immunoblot of c-FLIP level in four tumors from each NaCl- and PTX-treated group. **e**–**g** Number of CD31+ (**e**), Ki67+ (**f**), and cleaved caspase-3+ (casp-3c+) (**g**) cells/mm^2^ detected by immunohistochemistry (IHC) (three mice/group). Data are representative of two independent experiments. *P < 0.05. **h** Representative micrographs of cleaved caspase-3 staining by IHC in tumors. Arrows indicate positive cleaved caspase-3 cells. Scale bars: 100 μm

We next analyzed whether paclitaxel in combination with Poly(I:C) would have beneficial effects on tumor growth in vivo. Neither drug alone used at sub-optimal concentration (10 mg/kg for paclitaxel; 0.1 mg/kg for Poly(I:C)) had a significant effect on the growth of NSCLC NCI-H292 cells implanted into immunocompromised mice (Fig. 6a) and the survival of the mice was not improved (Fig. 6b). In contrast, paclitaxel/Poly(I:C) combination therapy inhibited the growth of the tumor and also significantly prolonged mice survival compared to either drug taken individually (Fig. 6a, b). Indeed, although all mice died or had to be sacrificed (tumor volumes >1800 mm^3^) after 17 days of treatment in NaCl-treated control condition, after 24 days in paclitaxel condition, or after 28 days in Poly(I:C) condition, more than 30% of the mice treated with paclitaxel/Poly(I:C) combination were still alive after 30 days of treatment. No overt toxicity was observed in Poly(I:C)-including treatment condition, and the body weight was not affected (Fig. 6c).

To determine the molecular mechanism of synergy, we first verified by immunoblotting that paclitaxel treatment efficiently reduced c-FLIP level in the tumors (Fig. 6d), as observed in vitro. Then, we found that the frequency of CD31+ or KI67+ cells was not reduced within the tumors by the combined treatment (Fig. 6e, f), suggesting that the synergy was not due to a reduction in tumor vascularization or cancer cell proliferation, respectively. Instead, the combined therapy was associated with an increase of the frequency of cleaved caspase-3+ apoptotic tumor cells (Fig. 6f, g). Interestingly, we found that even after repeated injections of Poly(I:C) in the mice, TLR3 expression in xenograft cancer cells was maintained (Supplemental Fig. 4). Altogether, these results suggest that paclitaxel and Poly(I:C) act synergistically to inhibit tumor growth in immunocompromised mice via the direct and selective induction of apoptosis by TLR3 in cancer cells.

## Discussion

Our data demonstrate that single release of either cIAPs or c-FLIP brake is sufficient to overcome resistance of human lung and oral cancer cell lines to TLR3-mediated apoptosis. However, we found that the release of the c-FLIP brake only results in the sensitization of all human cancer cell lines tested while the effects remain limited to a proportion of cell lines when the cIAPs brake only is released. In contrast, cIAPs and c-FLIP exert an efficient double brake in non-transformed cells, and thereby protect them against TLR3-mediated apoptosis. We took advantage of this knowledge to set up an effective and safe synergistic combination of TLR3 ligand with paclitaxel-mediated release of c-FLIP brake that could be translated to the clinic.

Our results suggest that cIAPs and c-FLIP brakes act on the same apoptotic pathway that depends on RIPK1. Combination of TLR3 ligand with smac mimetic molecules was previously proposed as cancer therapy based on pre-clinical murine cancer models^47^. Overcoming the resistance via cIAPs suppression probably depends on cancer types or cancer natural history because resistant melanoma cell lines or nasopharyngeal carcinoma latently infected by the Epstein-Barr virus can be sensitized to TLR3-induced apoptosis by the use of smac mimetics^7,8,10^. Moreover, triggering a RIPK1-mediated apoptotic and/or necroptotic death by smac mimetics against leukemia seems promising^48,49^. However, combination of TLR3 ligand with the release of c-FLIP brake should be considered as an alternative approach for triggering RIPK1-mediated apoptosis against resistant solid cancers that frequently lose the expression of RIPK3, a key protein in necroptosis signaling^45,46^. Accordingly, we did not find any evidence of necroptosis induction in response to TLR3 activation in our cellular models, which might be due to the very low level of RIPK3 detected in these cells.

Our data strongly suggest that the reduction of c-FLIP expression represents the main mechanism of synergy between Poly(I:C) and paclitaxel, as (a) paclitaxel pre-treatment reduced the expression of c-FLIP, but not cIAPs, which correlated with the sensitization to Poly(I:C) cytotoxicity in cancer cells, (b) c-FLIP overexpression abolished this synergy, and (c) cancer cell death remained strictly apoptotic. The resistance of non-transformed cells to the combination of TLR3 agonist with paclitaxel (and possibly other chemotherapies that downregulate c-FLIP in cancer cells, including irinotecan, gemcitabine, 5-fluorouracil, and oxaliplatin^42,50,51^), and the absence of toxicity in mice suggests that this strategy could be safe in patients as well. However, our data also predict the potential risk of combining TLR3 agonist with drugs that would compromise both cIAPs and c-FLIP, like etoposide^52^, as they might kill both tumor and normal cells.

The fact that non-transformed cells (HBEC3-KT and primary HBEC) and two cancer cell lines (NCI-H596 and SK-MES-1) did not exhibit a decrease of c-FLIP in response to paclitaxel is intriguing. However, it highlights that the effect of paclitaxel on c-FLIP level is not direct, and cannot be explained by the non-transformed vs. transformed state of the cells. Several reports indicate that paclitaxel-induced c-FLIP downregulation is controlled at the post-transcriptional level^40,41,53^. However, the molecular mechanism remains unclear as it can rely on the upregulation of the miR-512-3p microRNA^40^, or on the active degradation by the proteasome following sustained activation of JNK^53^. Interestingly, paclitaxel can also reduce the activation of STAT3 and, consequently, the expression of STAT3-dependent genes^54,55^. On the other hand, the expression of c-FLIP can be upregulated in a STAT3-dependent manner in response to MEK inhibitors in BRAF mutated colon cancer cells^56^, suggesting that c-FLIP level could also be regulated at the transcriptional level via STAT3 inhibition in response to paclitaxel treatment. Therefore, deciphering the precise molecular mechanism(s) of paclitaxel-induced c-FLIP downregulation represents an interesting future challenge that could lead to the identification of predictive markers of cell sensitivity to paclitaxel.

Our robust IHC protocol reveals that lung cancer is a promising target for TLR3 agonist, given that the expression of the receptor by tumor cells was reported to be a good biomarker for clinical response of breast cancer patients to Poly(A:U)^33^. Whether analysis of c-FLIP expression will improve the prediction of tumor sensitivity to TLR3 agonist alone is currently under investigation. According to our data, however, the combination of paclitaxel, a standard drug for lung cancer, with a TLR3 ligand should be effective against most TLR3+ cases, provided that they express caspase-8^57^. Lastly, the availability of clinical grade TLR3 ligand has long been an obstacle for the clinical translation of over a decade of encouraging pre-clinical data in mice. However, recent description of well-defined and homogeneous TLR3-specific ligands^58,59^ has revived the interest for targeting TLR3 in patients. In conclusion, the results presented here further support the targeting of TLR3+ squamous lung cancers with TLR3 ligand alone or in combination with c-FLIP-inhibitory chemotherapy.

## Methods

### Cell culture

All cell lines were purchased from ATCC (LGC Standards, Molsheim, France). NCI-H292 and NCI-H1703 cells were grown in RPMI 1640 medium (Life Technologies, Grand Island, NY, USA) supplemented with 10% fetal bovine serum, Hepes, NaPy, 100 U/ml penicillin/streptomycin, and 2 mM glutamine. NCI-H596 cells were grown in RPMI 1640 medium, supplemented with 10% fetal bovine serum and 100 U/ml penicillin/streptomycin. SKMES-1 cells were grown in MEM medium (Life Technologies), supplemented with 10% fetal bovine serum and 100 U/ml penicillin/streptomycin. SW900 cells were grown in Leibovitz’s L-15 medium (Life Technologies), supplemented with 10% fetal bovine serum and 100 U/ml penicillin/streptomycin. H314, H357, H400, and H413 cells were grown in DMEM: F12 (1:1) medium (Life Technologies), supplemented with 10% fetal bovine serum, 2 mM glutamine, 0.5 μg/ml hydrocortisone sodium succinate, and 100 U/ml penicillin/streptomycin. PE/CAPJ 15 cells were grown in IDMEM (Sigma-Aldrich, Saint-Louis, MO, USA) supplemented with 10% fetal bovine serum, 2 mM glutamine, and 100 U/ml penicillin/streptomycin. HT29 colon cancer cells were grown in McCoy’s medium (Life Technologies), supplemented with 10% fetal bovine serum, 2 mM glutamine, and 100 U/ml penicillin/streptomycin. Immortalized non-transformed HBEC3-KT cells were a gift from John D. Minna (Center for Therapeutic Oncology Research, Texas, US), and were grown in KSFM, supplemented with 2 ng/ml epidermal growth factor and 25 μg/ml bovine pituitary extract (Life Technologies). Primary HBEC cells were purchased from Lonza (Basel, Switzerland). They were grown in NHBE medium supplemented with 0.1 μg/ml retinoic acid (Lonza). All cell lines were tested for the absence of Mycoplasma after thawing and at the end of the culture by using a selective biochemical test (MycoAlert™ Mycoplasma Detection Kit, Lonza). Cells were used for experiments up to a maximum of 20 passages after thawing.

### Reagents and antibodies

Poly(I:C) (high molecular weight) was from Invivogen (San diego, CA, USA) and was used at 100 µg/ml unless otherwise stated. Z-VAD-FMK (R&D systems, Minneapolis, MN, USA) was used at 20 µM. Smac mimetic BV6 was first a gift from Ira Mellman and Wayne Fairbrother (Genentech, CA, USA), and later purchased from Selleckchem (Houston, TX, USA), and was used at 5 µM. Paclitaxel and necrostatin-1 were purchased from Sigma-Aldrich. RIPK3 kinase inhibitor GSK'872 was from Merck-Calbiochem (Darmstadt, Germany).

Antibodies were purchased from the following companies: anti-caspase-8 (clone C-20) (Santa CruzBiotechnology, Dallas, TX, USA), anti-actin (clone C4) (MP Biomedicals Europe, Illkirch, France), anticIAP1/2 (clone 315301) (R&D systems), anti-RIPK1 (clone 38) (BD Biosciences, San Jose, CA, USA), anti-FLIP (clone NF6) (Alexis Biochemicals, San Diego, CA, USA) anti-TRIF (Cell Signaling Technology, Danvers, MA, USA), anti-Bcl-xL (Cell Signaling), anti-TLR3 (cloneD10F10) (Cell Signaling) and clone 1210F1 (Dendritics, Lyon, France), anti-caspase-8 (clone 1C12) (Cell Signaling), anti-caspase-3 (Cell Signaling), anti-cleaved caspase 3 (clone 5A1E) (Cell Signaling), and anti-RIPK3 (clone E1Z1D) (Cell Signaling).

### Western blotting

Cells were lysed in cold lysis buffer (20 mM Tris-HCl (pH 7.4), 150 mM NaCl, 0.2% Nonidet NP40) supplemented with a protease inhibitor cocktail (Sigma-Aldrich) for 30 min on ice. Cell lysates were cleared by centrifugation (13,000×*g*, 15 min, 4 °C), and protein concentration was determined by the Bradford assay (Bio-Rad, Hercules, CA, USA). Proteins were resolved on SDS-PAGE, transferred onto PVDF membranes by electroblotting, and nonspecific binding sites were blocked using Tris-buffered saline containing 0.1% Tween-20 and 5% (w/v) dried milk or bovine serum albumin. After incubation with appropriate primary antibodies overnight, blots are incubated with secondary antibodies conjugated to horseradish peroxidase, then revealed using the ECL reagents.

### Cell transduction

The retroviral vectors pMSCV-puro-c-FLIP-long and pMSCV-puro-c-FLIP-short were used for the ectopic expression of c-FLIP long and c-FLIP short, respectively. Pseudotype viruses were generated from the packaging cell line GP2-293 from Clontech (Mountain View, CA, USA). Cells were transduced for 16 h with viral supernatants containing polybrene (8 mg/ml), washed in phosphate-buffered saline, and selected in complete medium containing puromycine (2.5 μg/ml) for 7 days.

### Cytokine measurement

Cytokine concentrations in cell supernatants were measured by ELISA, all performed in triplicates. Concentrations of RANTES, IL-6, and IP-10 were calculated using a cytokine enzyme immunoassay kit (R&D systems), according to the manufacturer’s recommendations. Absorbance was measured using the TECAN infinite 200 microplate reader (TECAN Group Ltd., Mannedorf, Switzerland).

### Quantification of cell survival and apoptosis

For cell survival measurement, 1 × 10^4^ cells (NCI-H292 and NCI-H1703) were seeded in 96-well plates and cultured for 24 h. The scheduled paclitaxel/Poly(I:C) treatment protocol was as follow: cells were treated for 2 h with Paclitaxel, washed, and 24 h later exposed to Poly(I:C) for 2 h. and then washed again; 48 h later, cell survival was measured with MTS assay (CellTiter 96 AQueous Non-Radioactive Cell Proliferation Assay reagents, Promega, Madison, WI, USA). Plates were incubated 2 h at 37 °C in the dark and absorbance was recorded at 470 nm and 690 nm using a Multiskan EX microplate photometer (Thermo Scientific, Pittsburg, PA, USA). A 690-nm wavelength absorbance was used to subtract the background.

For apoptosis measurement, cells were harvested by trypsinization and resuspended in binding buffer containing annexin V-FITC and PI (Abcys, Paris, France). FITC- and PI-labeled cell populations were analyzed by flow cytometry (FACS Calibur, Becton Dickinson, Franklin Lakes, NJ, USA) and CellQuest software (Bekton Dickinson), and expressed as percentage. Annexin V+ cells correspond to the total of Annexin V+ and Annexin V+/PI+ cells. For some experiments, cell death was measured with an Incucyte ZOOM® system (Essen BioScience, Michigan, USA) analyzing sytox green (SG)-positive cells. The 100% SG-positive cells were achieved by permeabilization of the cells using 0.02% Triton X-100, and the percentage of cell death was calculated as (induced SG+ cells−background SG+ cells) / (maximal SG+ cells−background SG+ cells) × 100.

### RNA interference

RIPK1 (J-004445-07) and control non-silencing (D-001810-03-20) siRNAs were from Dharmacon (ThermoFisher Scientific, Waltham, MA, USA). Caspase-8 (SI02661946) and c-FLIP (SI04951492) siRNAs were from Qiagen (Hilden, Germany). Synthetic TLR3 Stealth (TLR3HSS110816) was from ThermoFisher Scientific. Cells were transfected by using HiPerFect reagents (Qiagen) for 24–96 h. The final siRNAs concentrations varied from 5 nM to 100 nM according to cell lines and targeted proteins.

### Mice xenografts

Cells were subcutaneously injected into the flank of 6-week-old female BALB/c-nude mice (nine mice/group) (Charles River, Les Oncins, France). When the tumors reached 100 mm^3^, mice were treated once a week with paclitaxel (10 mg/kg), Poly(I:C) (0.1 mg/kg), or paclitaxel followed 24 h later by Poly(I:C). Tumor volume was measured every third day with a caliper. Mice were killed when their tumors reached 1800 mm^3^ or when they were moribund. Mice were housed in ANICAN, our specific pathogen-free animal facility.

### Immunohistochemistry

Immunostainings of human tumor specimen were performed on a Benchmark XT Ventana autostainer (Ventana Medical System, Tucson AZ, USA), using a three-step protocol and the Ventana detection kit UltraView DAB. For TLR3, tissue sections were incubated with rabbit polyclonal TLR3 antibody (PromoCell, Heidelberg, Germany) at the dilution 1:1500, with 90 min antigen retrieval in CC1 buffer. A cytoplasmic diffuse or granular immunostaining, with sometimes plasma membrane reinforcement, was considered as positive and scored using the product of the percentage of positive cells by the staining intensity, ranging from 0 to 3: 0 = “no staining”, 1 = “weak”, 2 = “moderate”, and 3 = “strong”. TLR3 immunohistochemistry was analyzed by Prof. S. Lantuejoul, pathologist at Centre Léon Bérard, Lyon, France.

Anesthetized mice were killed and tumors samples were fixed in 10% buffered formalin, embedded in paraffin, and then 3–4-µm-thick tumor sections were prepared. Immunostainings of mouse tumor specimen were performed on a Discovery XT Ventana autostainer (Ventana Medical System, Tucson AZ, USA), using the avidin–biotin complex protocol and the Ventana detection DabMap kit. Tissue sections were incubated with rabbit polyclonal anti-CD31 antibody (Anaspec, Fremont, CA, USA) at the dilution 1:200, or with rabbit monoclonal anti-Ki67 antibody (clone SP6, Spring bioscience, Pleasanton, CA, USA) at the dilution 1:200, or with rabbit monoclonal anti-cleaved caspase-3 antibody (clone 5A1E, Cell signaling, Beverly, MA, USA) at the dilution 1:200, with 40 min antigen retrieval in CC1 buffer. Image analysis was performed by using a light microscope (Eclipse E400, Nikon France, Champigny, France) equipped with a tri-CDD video camera (Sony, Japan), and a morphometric analysis software (Histolab, Microvision Instruments, Evry, France). The analysis of the number of CD31+, Ki67+, and cleaved caspase-3+ cells was performed blinded.

### Study approval

Human tissue samples were collected from surgical resection of lung tumors, and stored for scientific research in a biological resource repository (Centre de Ressources Biologiques, CHU Albert Michallon, Grenoble Hospital). National ethical guidelines were followed. All patients provided written informed consent. Tissue banking and research conduct was approved by the Ministry of Research (approval AC-2010-1129) and by the regional IRB (CPP 5 Sud Est).

Mice experiments were performed in accordance with European Union guidelines and validated by the local Animal Ethics Evaluation Committee (CECCAPP; NÅã CLB.2011.006).

### Statistics

For mice injected with NCI-H292 cells, Kaplan–Meier univariate survival analysis was performed, and *P*-values were calculated using the log-rank (Mantel–Cox) test. For TLR3 IHC analysis of human samples, Fisher exact test was performed for the number of positive cases data, and Mann–Whitney test for the median score data. For other statistical analysis, two-tailed unpaired *T*-test was used. *P < 0.05 and **P < 0.01 were considered as significant.

## Electronic supplementary material

Supplemental Figures
