# Supplementary material for: Release of c-FLIP brake selectively sensitizes human cancer cells to TLR3-mediated apoptosis
Source: Cell Death Dis. 2018 Aug 29;9(9):874. doi: 10.1038/s41419-018-0850-0 (PMC6115461; doi:10.1038/s41419-018-0850-0)
Supplement: Supplementary file 1 — Supplemental Figures [file 41419_2018_850_MOESM1_ESM.pdf]

Supplemental Figure legends

Supplemental Figure 1: Chemokines/cytokines secretion by cancer cells in response to Poly(I:C) treatment. Cancer cells were treated with Poly(I:C) (100 μg/ml) for 24h, and the concentrations of chemokines/cytokines in culture supernatants were determined by ELISA. Error bars represent S.E.M. of three independent experiments.

Supplemental Figure 2: Cell death induced by the BV6/siFLIP/Poly(I:C) combination in HBEC3-KT cells requires caspase-8 and RIPK1. a, c HBEC3-KT cells transfected with a control siRNA (siNS) or targeting caspase-8 (sicasp-8) (**a**) or targeting RIP1 (siRIP1) (**c**) were further treated with the siFLIP/BV6 combination, and then exposed to Poly(I:C) (100 μg/ml) for 6h. The percentage of Annexin V+ cells was determined by flow cytometry. Error bars represent S.E.M. of three independent experiments. $^{**}P<0.01$. **b, d** Validation by western blot of the sicasp-8 and siRIP1 efficiency in HBEC3-KT cells. **e** HBEC3-KT cells were treated with the siFLIP/BV6 combination in presence or absence of Z-VAD (20 μM) or necrostatin-1 (nec-1) (2.5 μM), and then exposed to Poly(I:C) (100 μg/ml) for 6h. The percentage of Annexin V+ cells was determined. Error bars represent S.E.M. of at least two independent experiments.

Supplemental Figure 3: Paclitaxel chemotherapy decreases c-FLIP expression and sensitizes cancer cells to TLR3-mediated death. a Analysis by western blot of c-FLIP level in NCI-H1703 cells treated with 250 nM paclitaxel (PTX) for 2h, washed, and then incubated with medium for 24h. **b** Viability curves of NCI-H1703 cells treated with increasing doses of PTX for 2h, washed, and 24 h later exposed to increasing doses of Poly(I:C) for 2 h, and then washed. 48 h later, cell survival was measured with MTS assay. Error bars represent S.E.M. of three independent experiments. $^{*}P<0.05$ and $^{**}P<0.01$ *versus* Poly(I:C)-treated cells. **c** Combination index (CI) for a fraction affected (Fa) of 50, 75, or 90% of the drug association between PTX and Poly(I:C) for NCI-H1703 cells and calculated using the method of Chou and Talalay. Synergy is characterized by a CI<0.9, additivity by a CI=1 +/- 0.1, and antagonism by a CI >1.1. Error bars represent S.E.M. of three independent experiments. **d** Percentage of Annexin V+ NCI-H1703 cells treated with 250 nM PTX as in (**a**), and then exposed or not 24h later to sub- optimal concentration of Poly(I:C) (0.08 μg/ml) for 6h. Error bars represent S.E.M. of three independent experiments. **e** Analysis by western blot of c-FLIP level in H400 cells treated with PTX as in (**a**). **f, g** Percentage of Annexin V+ H400 cells treated with 250 nM PTX as in (**a**), and then exposed to increasing doses of Poly(I:C) for 6h (**f**), in presence or absence of Z-VAD (20 μM) (**g**). Error bars represent S.E.M. of two independent experiments. **h, j** Percentage of Annexin V+ SK-MES-1 (**h**) and NCI-H596 (**j**) cells treated with 250 nM PTX as in (**a**), and then exposed to Poly(I:C) (100 μg/ml) for 6h. Error bars represent S.E.M. of two independent experiments. **i, k** Analysis by western blot of c-FLIP levels in SK-MES-1 (**i**) and NCI-H596 (**k**) cells treated with 250 nM PTX as in (**a**).

Supplemental Figure 4: TLR3 expression in NSCLC xenografts

TLR3 expression by immunohistochemistry in NCI-H292 xenografts tumors at the sacrifice of the mice treated with paclitaxel alone (left panel) or in combination with Poly(I:C) (right panel). Scale bars: 100 μm.

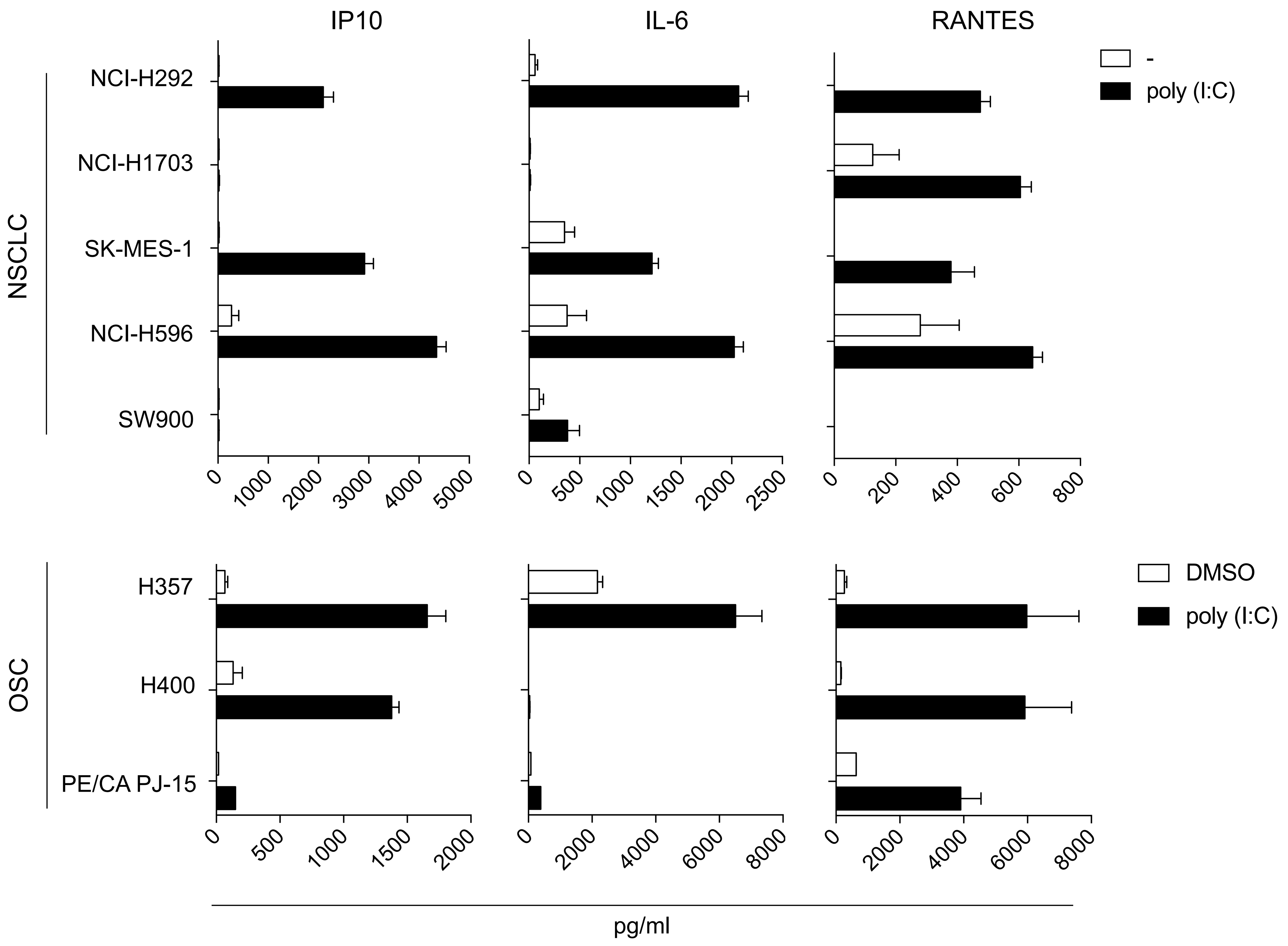

Supplemental 1

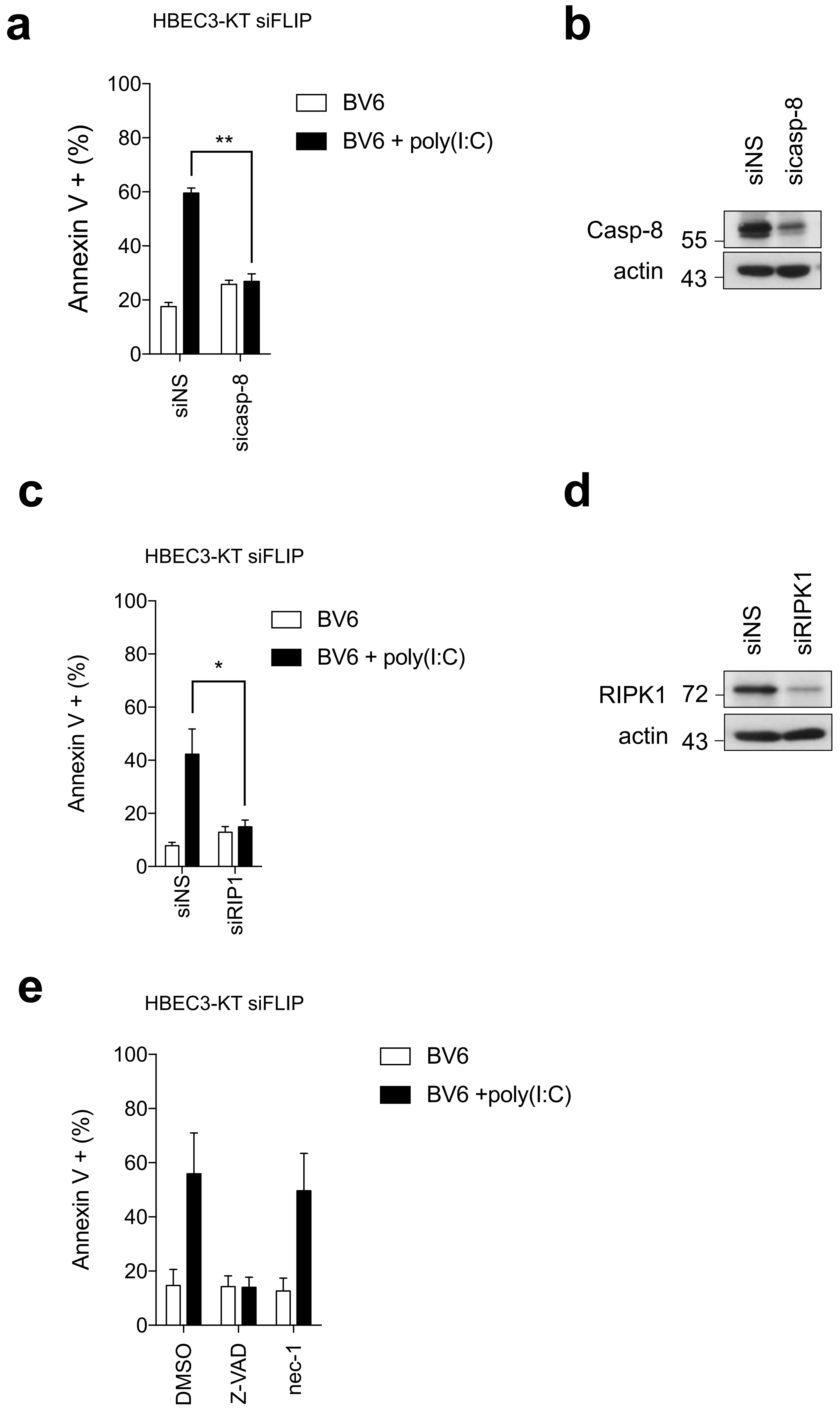

Supplemental 2

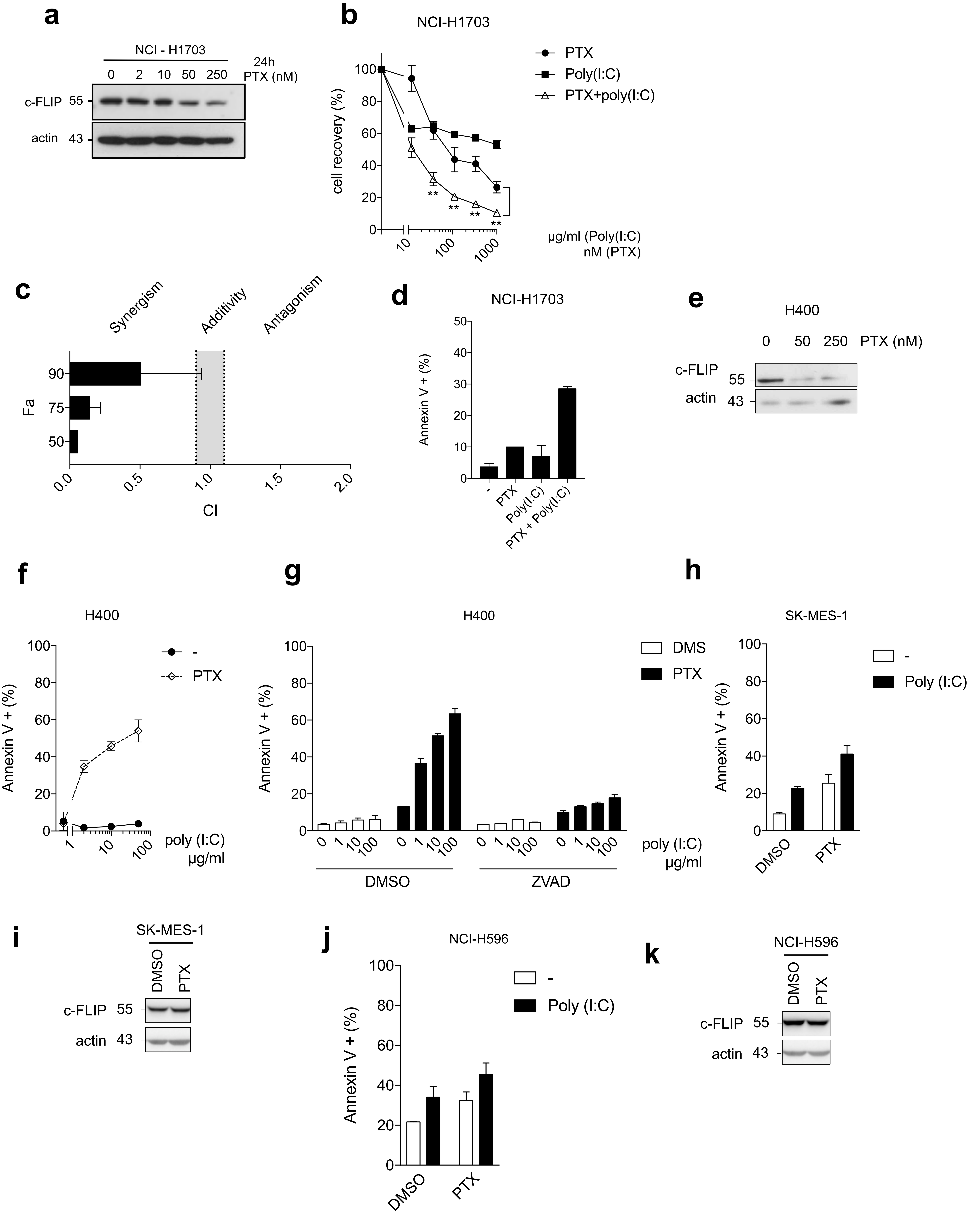

Supplemental 3

Paclitaxel | Paclitaxel + Poly (I:C)

TLR3

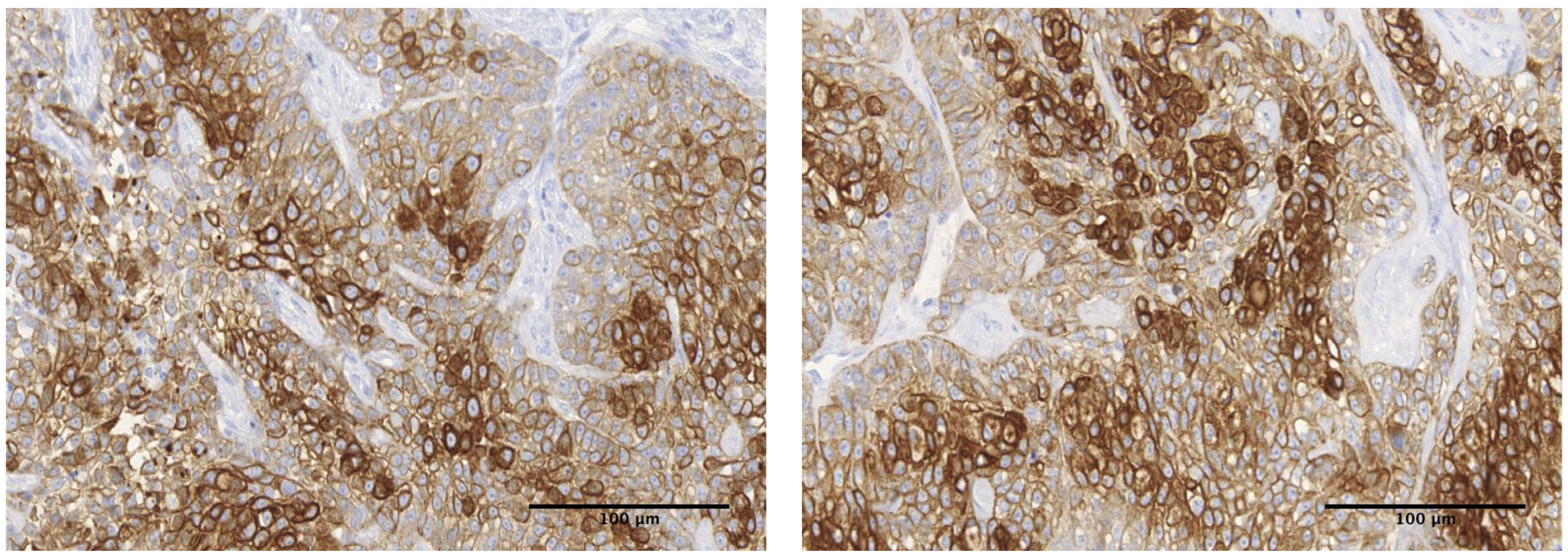

Supplemental 4
